# Rotor strength and critical speed analysis of a vertical long shaft fire pump connected with different shaft lengths

**DOI:** 10.1038/s41598-022-13320-z

**Published:** 2022-06-07

**Authors:** Haiqin Song, Jinfeng Zhang, Fan Zhang

**Affiliations:** National Research Center of Pumps, Jiangsu University, Zhenjiang, 212013 China

**Keywords:** Energy science and technology, Engineering, Materials science

## Abstract

The vertical long shaft fire pump (VLSFP) is mainly used in fire-fighting places far away from land and lacking large amounts of water supply. The paper selected the XBC18-178-240LC3 model of VLSFP as the research object. First, the experimental–numerical hydraulic performance of the single-VLSFP was carried out, and then the hydraulic performance of the multi-VLSFP was analyzed by the same numerical simulation method as single-VLSFP. After that, three rotor models (Z4 model, Z5 model-original model and Z6 model) were designed by modeling software, connected by different length and number of the shaft section under the same total length of the intermediate shafts. Finally, the rotor’s strength and critical speed of three models were analyzed and checked via the CFD simulation and the Workbench software. The study mainly found: (1) Through the strength check of the impeller, maximum equivalent stress of the three models was less than the allowable stress of the rotor material, which indicated the structural design of them met the safety requirement; (2) Through the critical speed check of the shafting rotor, the working speed of the VLSFP was lower than 0.8 times the first-order critical speed of the three models, which indicated the rotor could avoid the resonance and the structure of the three models met the dynamic design requirement. According to the stress check of the impeller and the critical speed check of the shafting rotor, combining the time and labor cost when the VLSFP was installed and disassembled many times before and after the test or operation, the paper selected the Z4 model to be the optimal model, which could provide a theoretical support for the subsequent structure design optimization of the vertical long shaft fire pump.

## Introduction

The vertical long shaft fire pump (VLSFP), mainly used in fire-fighting places far away from land and lacking large amounts of water supply, such as offshore platforms and wharfs, works by taking the seawater as the fire-fighting water source. It has the advantages of small footprint, large flow, high lift, fast start-up. Compared with traditional pumps, the shaft of VLSFP is extraordinarily long and composes of many shaft sections. In addition, the length of the drive shaft can be adjustable according to the sea level. When the sea level is lower than the installation of the pump system, the VLSFP can invert the water to avoid problems such as water diversion and cavitation caused by the higher suction height. As a large vertical rotating machine, the stability of its rotor system is the key to the safety of the pump system. If the working speed of the pump crosses or approaches the critical speed, the rotor system will vibrate^1,2^.

The rotor dynamics analysis methods are mainly based on the transfer matrix method and the finite element method. The transfer matrix method was proposed by Prohl^3^, and then improved by Horner and Pilkey^4^. Since then, extensive research has been conducted on it^5–8^. However, due to excessive simplification to the rotor by the transfer matrix method, it is difficult to ensure the computational accuracy of the model. In comparison, the finite element method can process the complicated model and calculation^9,10^. Therefore, the finite element method has become the preferred method for rotor dynamics analysis. In addition, in actual projects, the blades of rotating machinery are prone to cracks during long-term operation. Many factors will affect blade fatigue failures, including material, structure, processing technology, temperature, pressure, external shock and so on^11–15^.

In rotor dynamics, modal analysis and critical speed are also research focus. Chivens and Nelson^16^, Heydari and Khorram^17^, and She et al.^18,19^ studied the influence of the disk's flexibility on the critical speed and natural frequency of a rotating shaft-disk system. Taplak and Parlak^20^ built the model of a gas turbine rotor and adopted Dynrot program to obtain the Campbell diagram and the critical speed of the rotating systems for investigating dynamic behaviors of rotors. Castillo et al.^21^ confirmed that the impact test was a useful method for modal parameters identification of electrical submersible pump. Minette et al.^22^ investigated the dynamic behavior of an electrical submersible pump under operational conditions installed in a test well by identification of its natural frequency and damping parameters, using the Least Square Complex Exponential method. Huang et al.^23^ studied the modeling method of the rotor blade modes of the turbo-molecular pump, proposing a simplified method of the blade modified model based on the basic invariance principle of the mass and the moment of inertia before and after simplification.

However, there is very little literature about the analysis of the rotor modal and critical speed of the shafting of the VLSFP. In addition, in practical engineering project, the VLSFP, which is connected with more shaft segments, needs to take more time and the labor cost when it is installed and disassembled many times before and after the test or operation. Therefore, the paper selected the XBC18-178-240LC3 VLSFP as the research object. First, the experimental–numerical hydraulic performance of the single-VLSFP was carried out, and then the hydraulic performance of the multi-VLSFP was analyzed by the same numerical simulation method as single-VLSFP. After that, three rotor models (Z4 model, Z5 model and Z6 model) were designed by modeling software, connected by different length and number of the shaft section under the same total length of the intermediate shafts. Finally, the rotor’s strength and critical speed of three models were analyzed and checked via the CFD simulation and the Workbench software, and the optimal solution was selected from the three models, providing a theoretical support for the subsequent design optimization of the vertical long shaft fire pump.

## Numerical methods

### Model introduction

**Figure 1:**
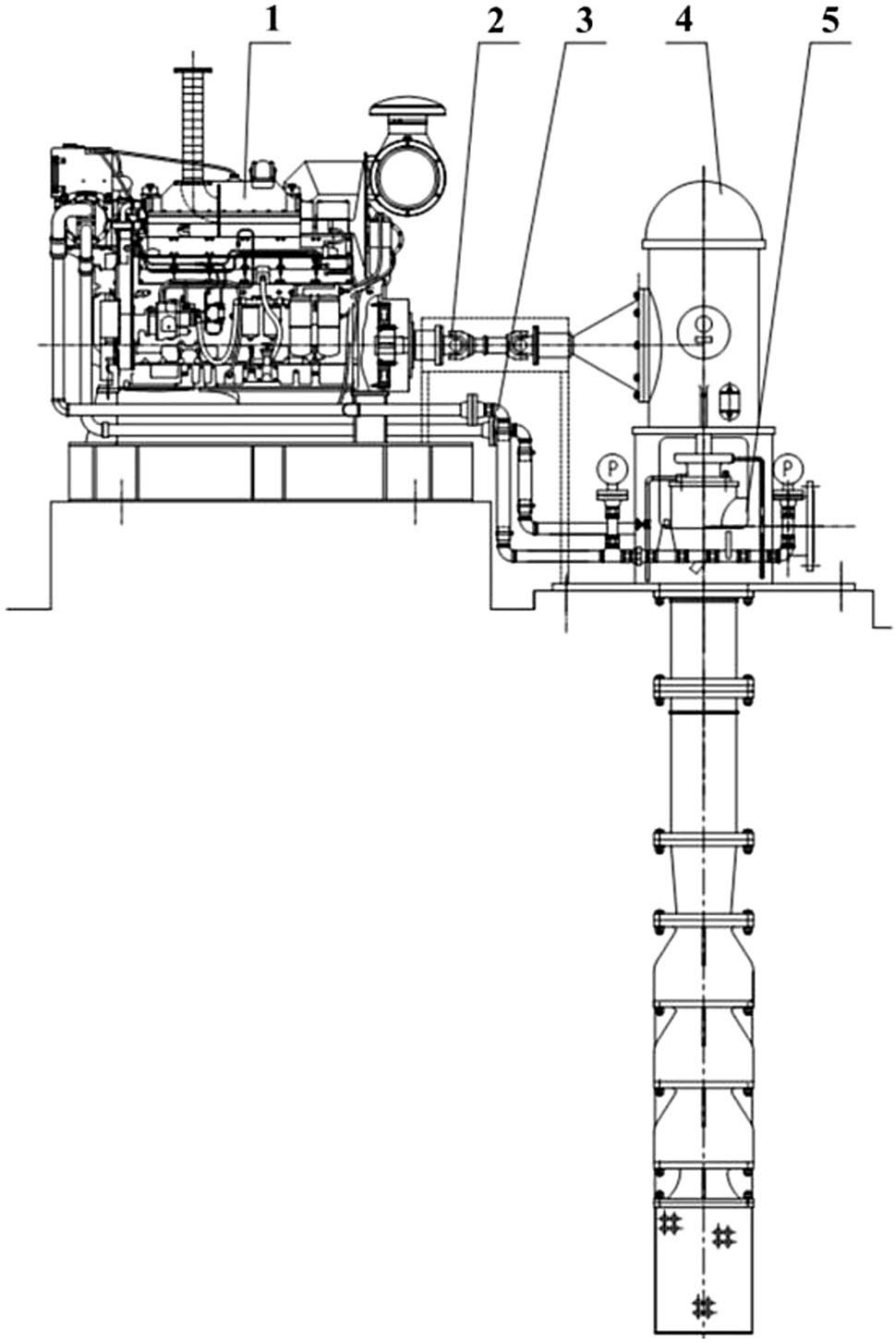
Overall structure of the XBC18-178-240LC3 VLSFP. Annotation: 1 Diesel engine; 2 Coupling and drive shaft; 3 Cooling water pipe; 4 Gear box; 5 VLSFP.

In Table 1, the key design parameters of the XBC18-178-240LC3 VLSFP are shown and in Fig. 1, the drawing of the overall pump is shown. For the VLSFP part, its integral pump shaft consists of 7 single shafts (1 section of impeller shaft, 5 sections of intermediate shaft and 1 section of drive shaft) connected by sleeve couplings. In terms of length, the impeller shaft is 2001 mm, the intermediate shaft is 1848 mm (5 sections are the same), the transmission shaft is 2232 mm, and the shaft section gap is 2 mm, resulting in the total of 13,485 mm for the pump shaft. The material of the pump shaft and impeller is duplex stainless steel (00Cr22Ni5Mo3N), which has the following properties: density = 7850 kg/m^3^, elastic modulus = 2.0 × 10^11^ Pa, Poisson’s ratio = 0.3, tensile strength = 620 MPa, yield strength = 450 MPa, and allowable stress = 250 MPa.

**Table 1:** Basic parameters of the VLSFP model.

Design parameters	Values
Flow rate (*Q*_d_)	864 m^3^/h
Single-head (*H*)	43.3 m
Operation speed (*n*)	1485 r/min
Number of impeller blades	6
Diameter of impeller inlet (*D*_j_)	209 mm
Diameter of impeller outlet (*D*_2_)	383 mm
Width of impeller blade outlet (*b*_2_)	58.7 mm
Number of guide vanes	5
Diameter of guide vane inlet (*d*_j_)	458.8 mm
Diameter of guide vane outlet (*d*_2_)	209 mm
Outlet width of guide vane (*b*_3_)	55.5 mm
Number of pump shaft sections	7
Total length of pump shaft	13,485 mm

### Three-dimensional modeling

**Figure 2:**
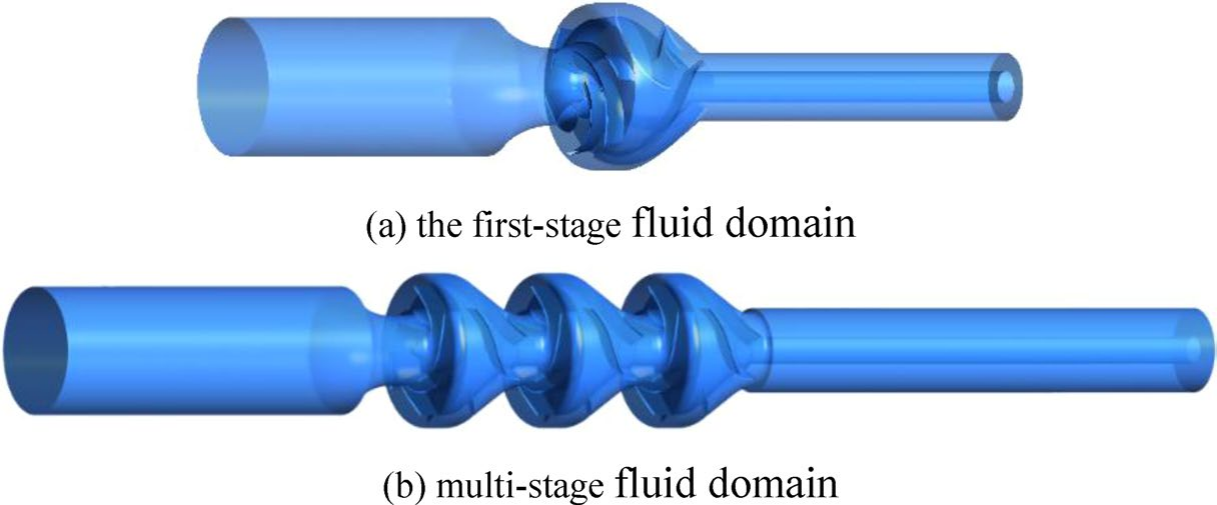
Three-dimensional fluid domains of the VLSFP.

The paper uses Creo5.0 software to carry out three-dimensional modeling of the first- and multi-stage fluid domains of the VLSFP, based on its actual size. The fluid domain refers to the water body flowing through each part of the pump, which is the reason that its shape is similar to the structure of the pump. As shown in Fig. 2, the first-stage fluid domain includes the water bodies flowing through the inlet, first-stage impeller, first-stage space guide vane and outlet, and the multi-stage fluid domain includes the water bodies flowing through the inlet, three impellers, three space guide vanes and the outlet.

### Computational domain meshing and independence analysis

**Figure 3:**
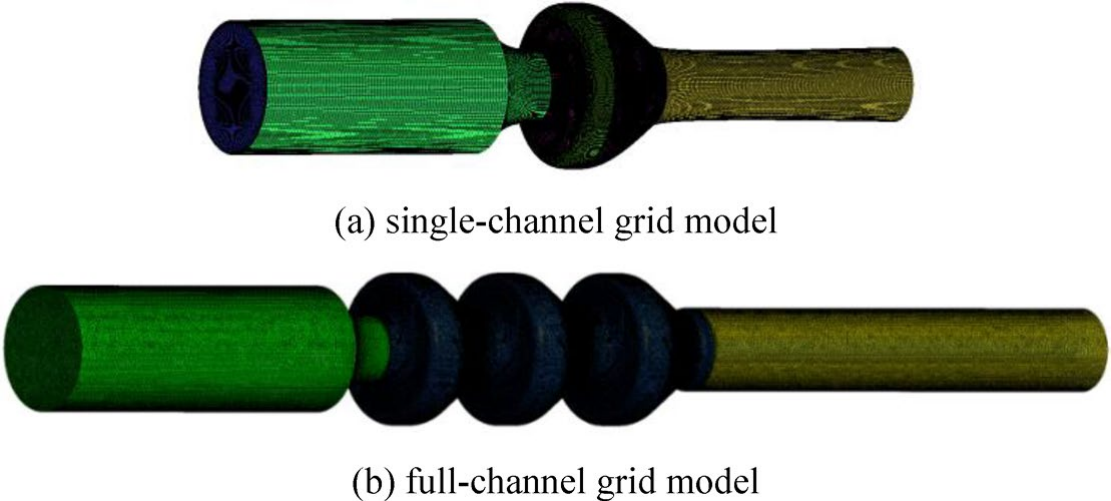
Grid of calculation domains.

**Figure 4:**
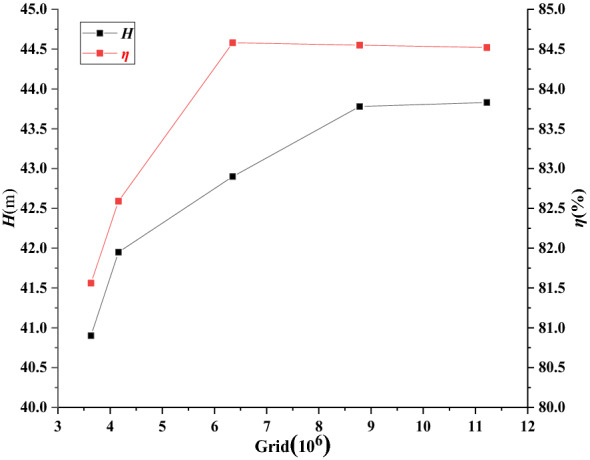
Grid independence test.

Considering the geometric characteristics, computing resources and accuracy of the water model, the ANSYS ICEM17.0 software is adopted to mesh the single channel and full channel of the VLSFP, and the boundary layer is processed to ensure the number and quality requirements of grids. Figure 3a is the single-channel grid model, and Fig. 3b is the full-channel grid model. Figure 4 shows the 5 groups of grid independence analysis results of the single-VLSFP model. As shown in Fig. 4, when the number of grids exceeded 8,782,000, the head and efficiency of the single-VLSFP tend to be stable. Therefore, considering the calculation accuracy and time, the fourth group of grids (8,782,000) is finally selected.

### Boundary conditions and solution settings

The steady numerical simulation of the VLSFP internal flow field is carried out by the ANSYS CFX17.0 software. The followings are the settings: water is used as the medium; the RNG k-ε turbulence model is adopted; the dynamic and static interface is set to Frozen Rotor mode; the boundary conditions are set to pressure inlet and mass flow outlet; the reference pressure selects a standard atmospheric pressure; the automatic wall function is selected to process the near the wall area that is set as a smooth wall; the solution discretization is set to the second-order upwind style; and the convergence residual is set to 10^–4^.

### Hydraulic performance verification

**Figure 5:**
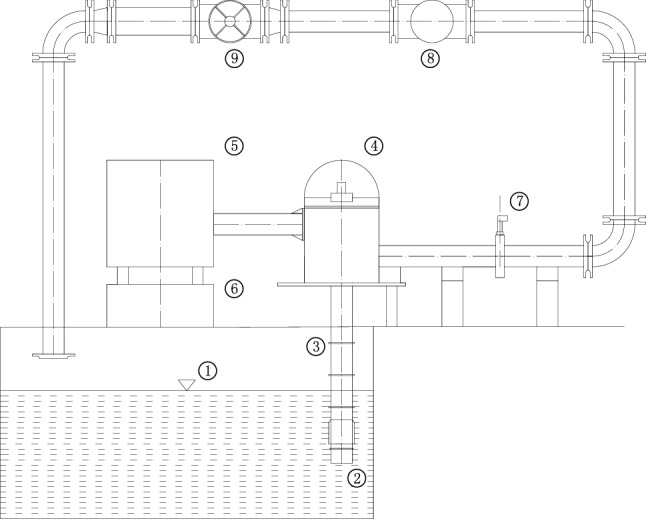
Experimental setup of the single-VLSFP. Annotation: 1 Pool; 2 Filter screen; 3 VLSFP; 4 Gear box; 5 Diesel engine; 6 Diesel engine base; 7 Pressure gauge; 8 Electromagnetic flow-meter; 9 Throttle.

The external characteristic test of the single-VLSFP is carried out in the open test bench of Kunshan Pudong Fluid Equipment Co., Ltd., Suzhou City, Jiangsu Province. It is found that the error of efficiency and head measurements is less than 2%. In this paper, the numerical calculation of the hydraulic performance of single-VLSFP is carried out and compared with the experimental data. After obtaining the reliable calculation method for single-VLSFP, the same numerical settings are applied to the multi-VLSFP. The schematic diagram of the single-VLSFP experimental setup is presented in Fig. 5.

#### Hydraulic performance of the single-VLSFP

**Figure 6:**
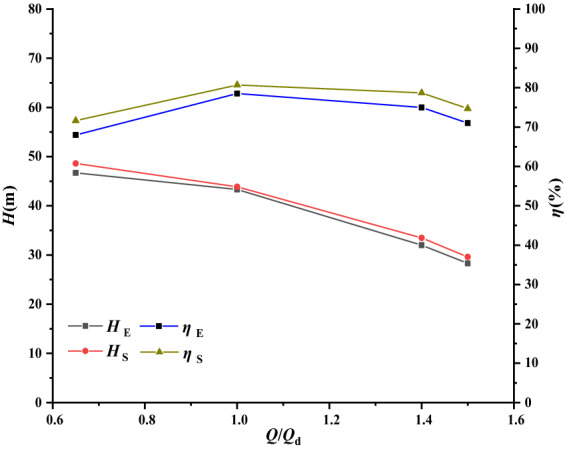
Performance characteristics of the single-VLSFP.

The hydraulic parameters of the pump mainly refer to flow rate, head, efficiency and so on, which can reflect the hydraulic performance of the pump. The hydraulic performance parameters (experimental and numerical) of the single-VLSFP under four working conditions of 0.65*Q*_d_, 1.0*Q*_d_, 1.4*Q*_d_ and 1.5*Q*_d_ are shown in Table 2. Here, *Q*_d_ refers to the flow rate at rated operating condition; *H*_E_, *H*_s_, ${\varepsilon }_{H}$, ${\eta }_{E}$, ${\eta }_{S}$ and ${\varepsilon }_{\eta }$ in Table 2 are respectively head under experiment, head under simulation, relative error of the head, efficiency under experiment, efficiency under simulation and relative error of the efficiency. As shown in Table 2, the relative error of the head and efficiency at the rated operating point are 1.27% and 2.78% respectively. Further away from the rated operating point, the relative errors of the head and efficiency increase, but the maxima are only about 5%, which is within the normal error range. Figure 6 provides a comparison of the hydraulic performance curve obtained by experiment and simulation for the single-VLSFP. It showed the data agreement is perfect. The above results verify that the numerical simulation method is reliable and can be used to predict the internal and external characteristics of the single-VLSFP.

**Table 2:** Hydraulic performance parameters of the single-VLSFP.

*Q*/*Q*_d_	*H*_E_/m	*H*_S_/m	${\varepsilon }_{H}$/%	${\eta }_{E}$/%	${\eta }_{S}$/%	${\varepsilon }_{\eta }$/%
0.65	46.7	48.59	4.05	68	71.635	5.345
1.0	43.3	43.85	1.27	78.5	80.686	2.78
1.4	32	33.47	4.59	75	78.684	4.912
1.5	28.3	29.61	4.63	71	74.692	5.2

#### Hydraulic performance of the multi-VLSFP

**Figure 7:**
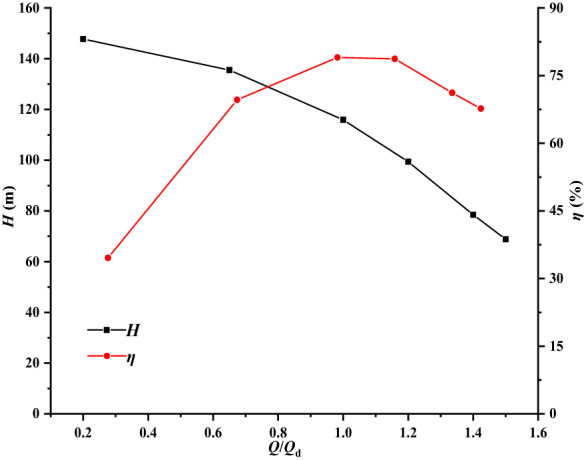
Hydraulic performance curve of multi-VLSFP.

The hydraulic performance of the multi-VLSFP is analyzed to check the structural rigidity and strength of the rotor under pre-stress. Six working conditions, i.e., 0.2*Q*_d_, 0.65*Q*_d_, 1.0*Q*_d_, 1.2*Q*_d_, 1.4*Q*_d_ and 1.5*Q*_d_, are calculated by the same numerical simulation method as single-VLSFP. Table 3 shows the simulated hydraulic performance. With reference to Table 2, the simulated head of the multi-VLSFP is much larger than (about 2.3–2.8 times) that of the single-VLSFP under the same working condition, which corresponds with the actual situation. As shown in Fig. 7, the simulated efficiency of the multi-VLSFP is slightly lower than that of the single-VLSFP, which is also in line with the actual situation of the project.

**Table 3:** Predicted hydraulic performance values of the multi-VLSFP.

*Q*/*Q*_d_	*H*/m	*η*/%
0.2	147.758	34.5758
0.65	135.543	69.6283
1.0	115.892	79.0326
1.2	99.4021	78.7308
1.4	78.4629	71.193
1.5	68.8573	67.6871

## Rotor strength check

### Rotor model introduction

**Figure 8:**
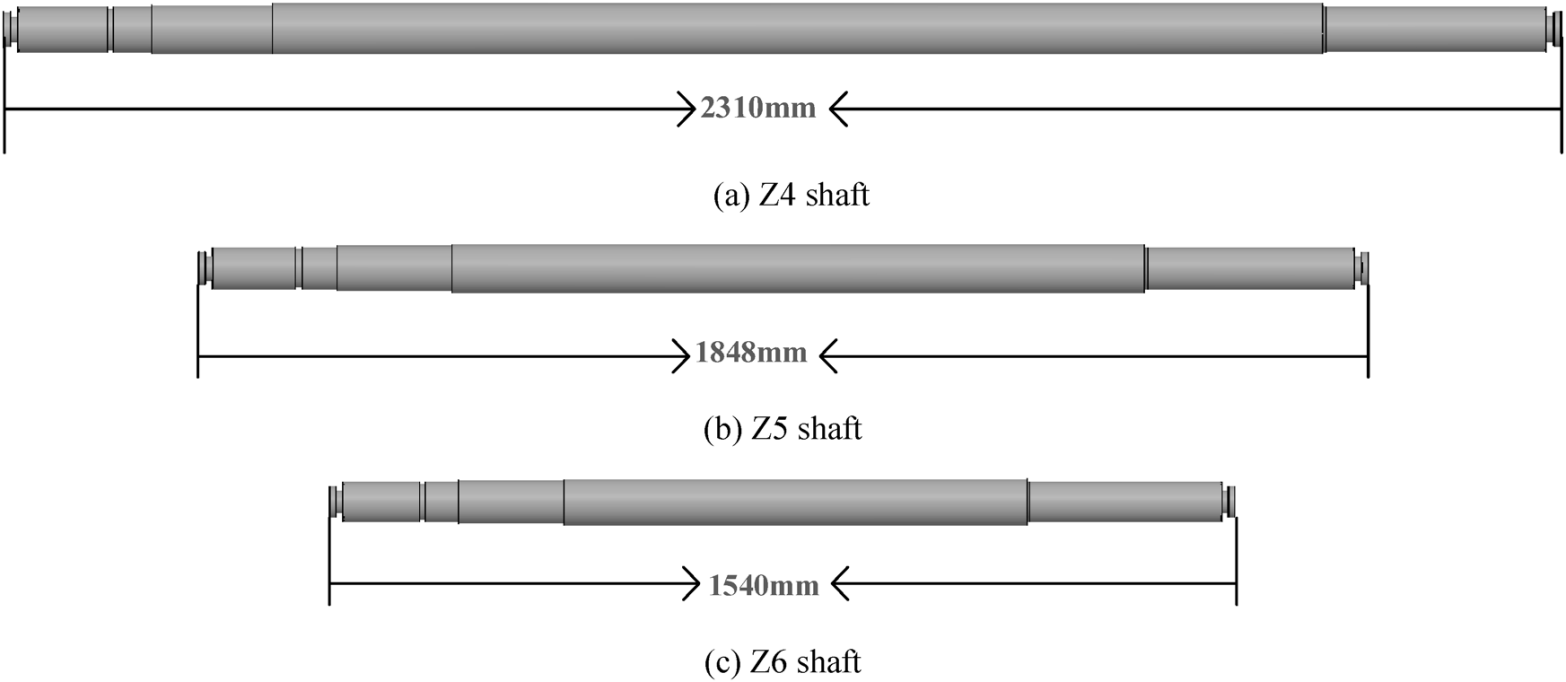
Three single-segment intermediate shafts: Z4, Z5 and Z6.

This study designs three connection schemes of different shaft lengths by modeling software. The keyway, sleeve couplings and other parts are simplified accordingly on the basis of not affecting the basic structure of the shafting rotor. Such a way can reduce the number of grids of the shafting rotor under the premise of ensuring a higher grid quality to shorten the calculation time. Combining the original shaft design of the XBC18-178-240LC3 model and the actual processing capacity of the pump factory, three single-segment intermediate shafts denoted as Z4, Z5 and Z6 shafts are shown in Fig. 8. Each part of three intermediate shafts is the same, apart from the length of the middle part.

**Figure 9:**
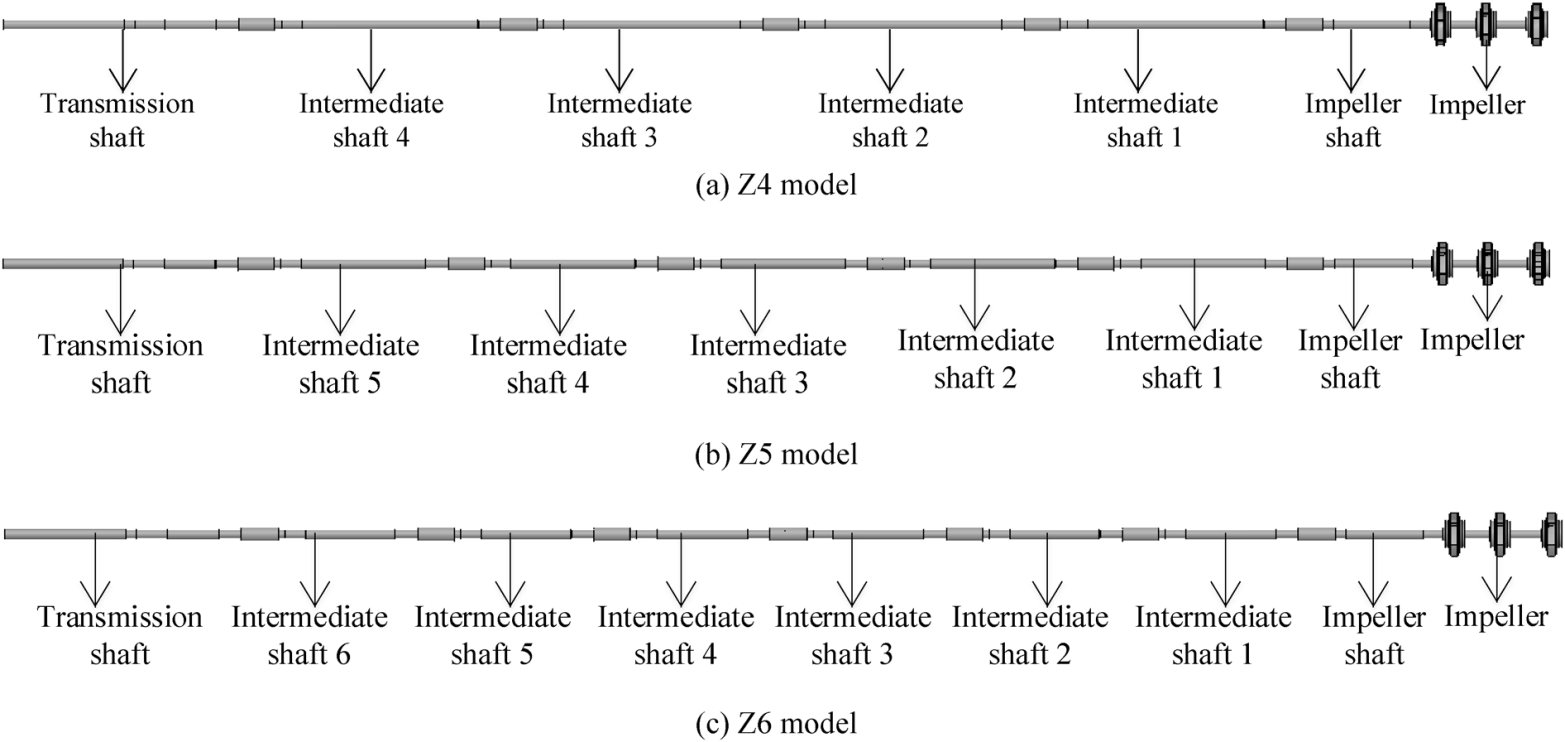
Three models of the VLSFP shafting rotor.

Figure 9b is the original model of the VLSFP shafting rotor, which consists of 7 single shafts (1 impeller shaft, 5 intermediate shafts and 1 drive shaft) through the sleeve couplings, and the design parameters of each part are introduced in detail in “Model introduction” section. This model is referred to as Z5 model, since it has 5 intermediate shafts. Similarly, Fig. 9a,c show the models with 4 and 6 intermediate shafts, referred to as Z4 and Z6, respectively. All parts of shafting rotor are the same in the three models, except for the length and number of the single-intermediate shaft, and the total length of the shafting rotor remains unchanged.

### Boundary conditions setting

ANSYS Workbench18.0 is used to perform statics analysis of the rotor. It is necessary to set the boundary conditions of the rotor according to actual conditions. The boundary conditions refer to the loads and supports. For the VLSFP of this paper, three kinds of loads are considered: the fluid force, the gravitational force and the centrifugal force. The fluid force is loaded to realize unidirectional fluid–structure interaction. Here, the fluid–structure interaction refers to a multi-physical field coupling between the laws describing fluid dynamics and structural mechanics. When a flowing fluid contacts with a solid structure, the structure is subjected to the stresses and strains, and these forces deform the structure. In addition, the gravitational force is loaded by adding a vertical downward gravitational acceleration, and the centrifugal force is loaded by adding the rotation speed. For the choice of constraints, since the shafting rotor is cylindrical, the “Cylindrical Supports” are loaded on the bearing’s locations to limit the axial and radial displacement of the rotor. Besides, the “Remote Displacement” is loaded on the top surface of the drive shaft to restrict the rotation of the rotor, except the axial rotation.

**Figure 10:**
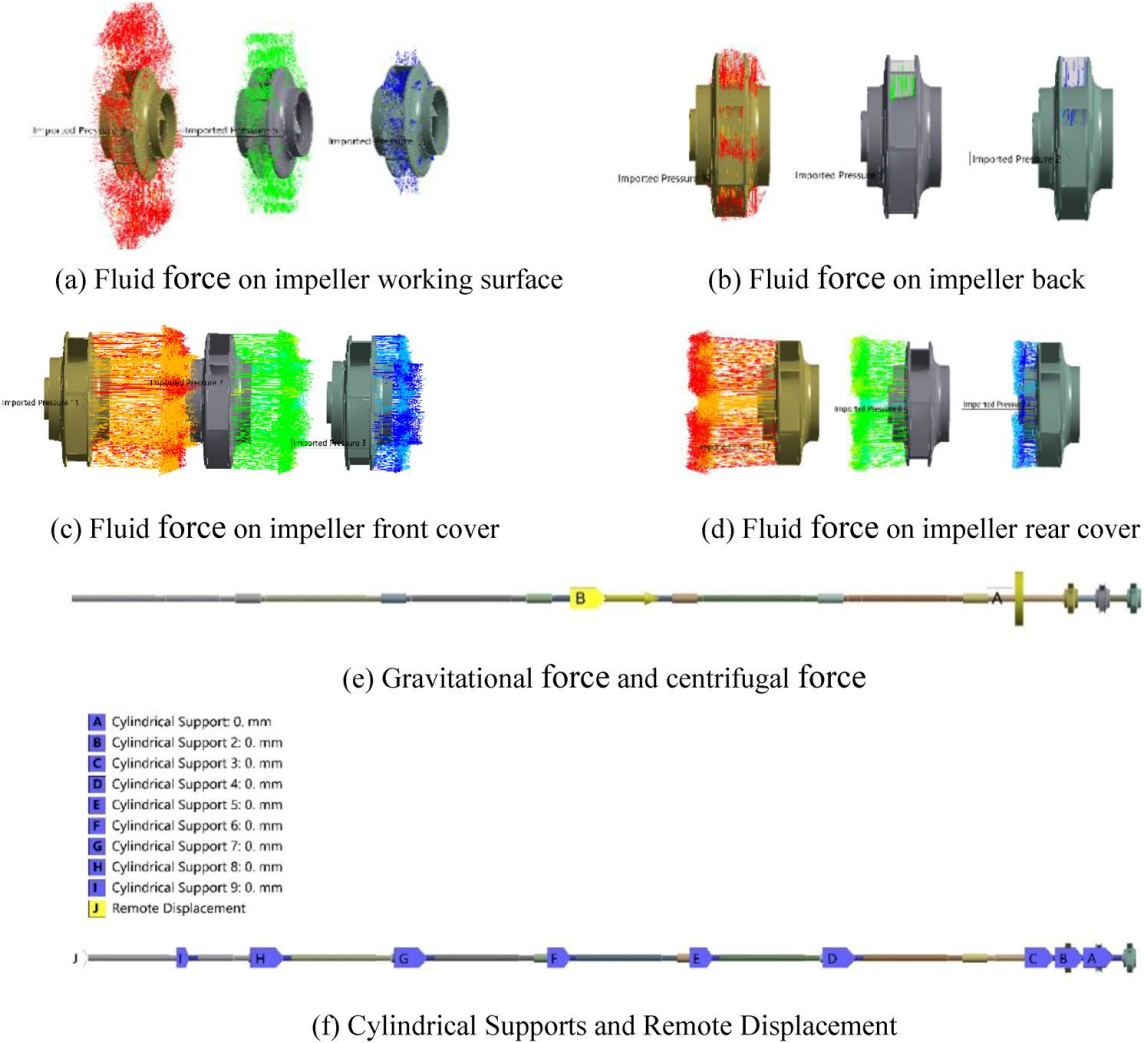
Setting of the boundary conditions.

Taking the original model (Z5) as an example. Figure 10a–d show the fluid force, and Fig. 10e shows the gravitational force and centrifugal force, where “A” represents the centrifugal force and “B” represents the gravitational force. Figure 10f shows the support setting of the rotor model, in which the “Cylindrical Supports” are represented by the letters “A-I” and the Remote Displacement is represented by the letter “J”.

The settings of the above loads and supports effectively ensure the consistency between the static calculation and the actual operating state of the rotor. In addition, factors such as gyro torque and sudden load changes have little effect on the results of rotor statics, which are not considered in this paper.

### Rotor deformation

**Figure 11:**
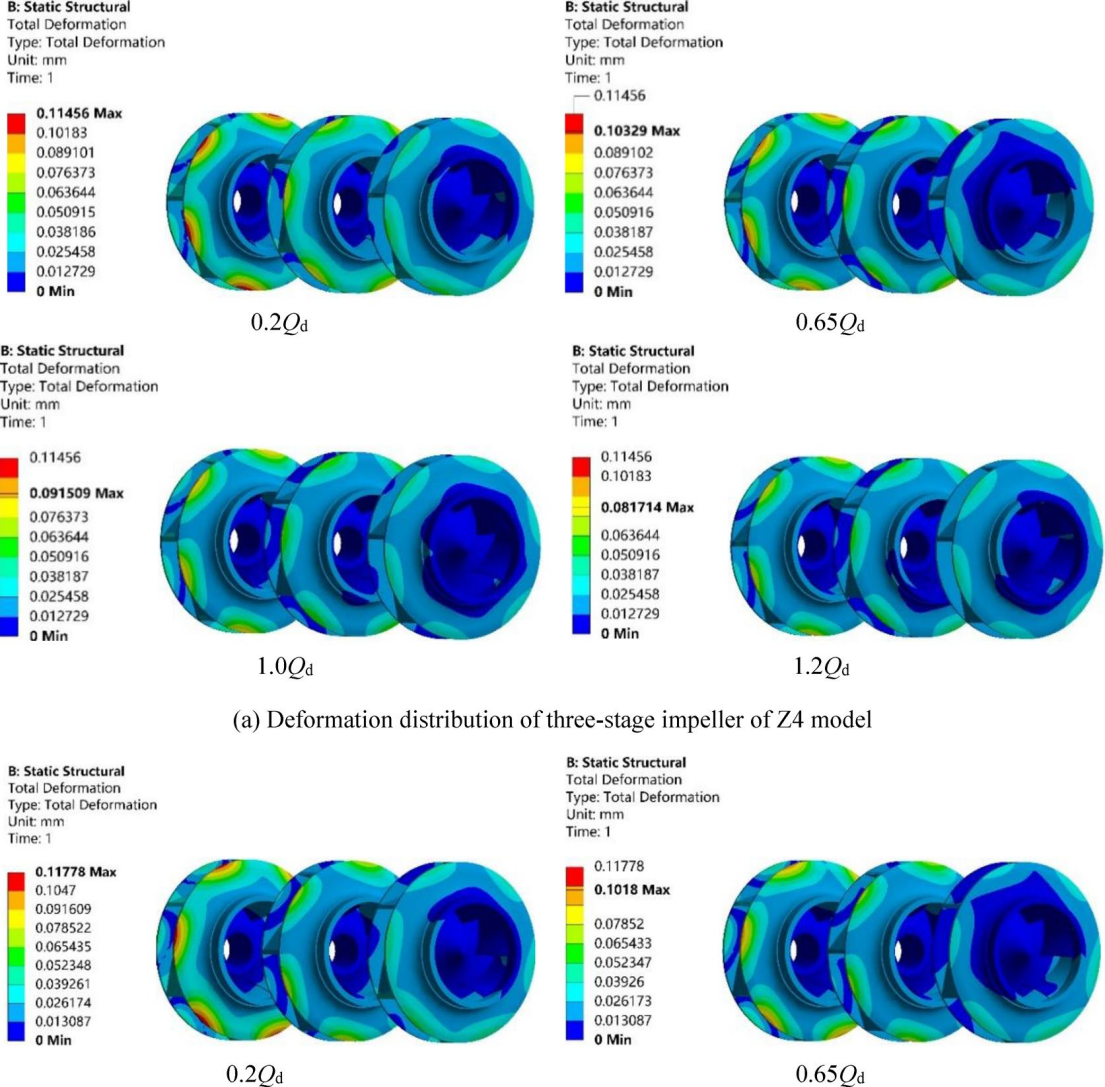

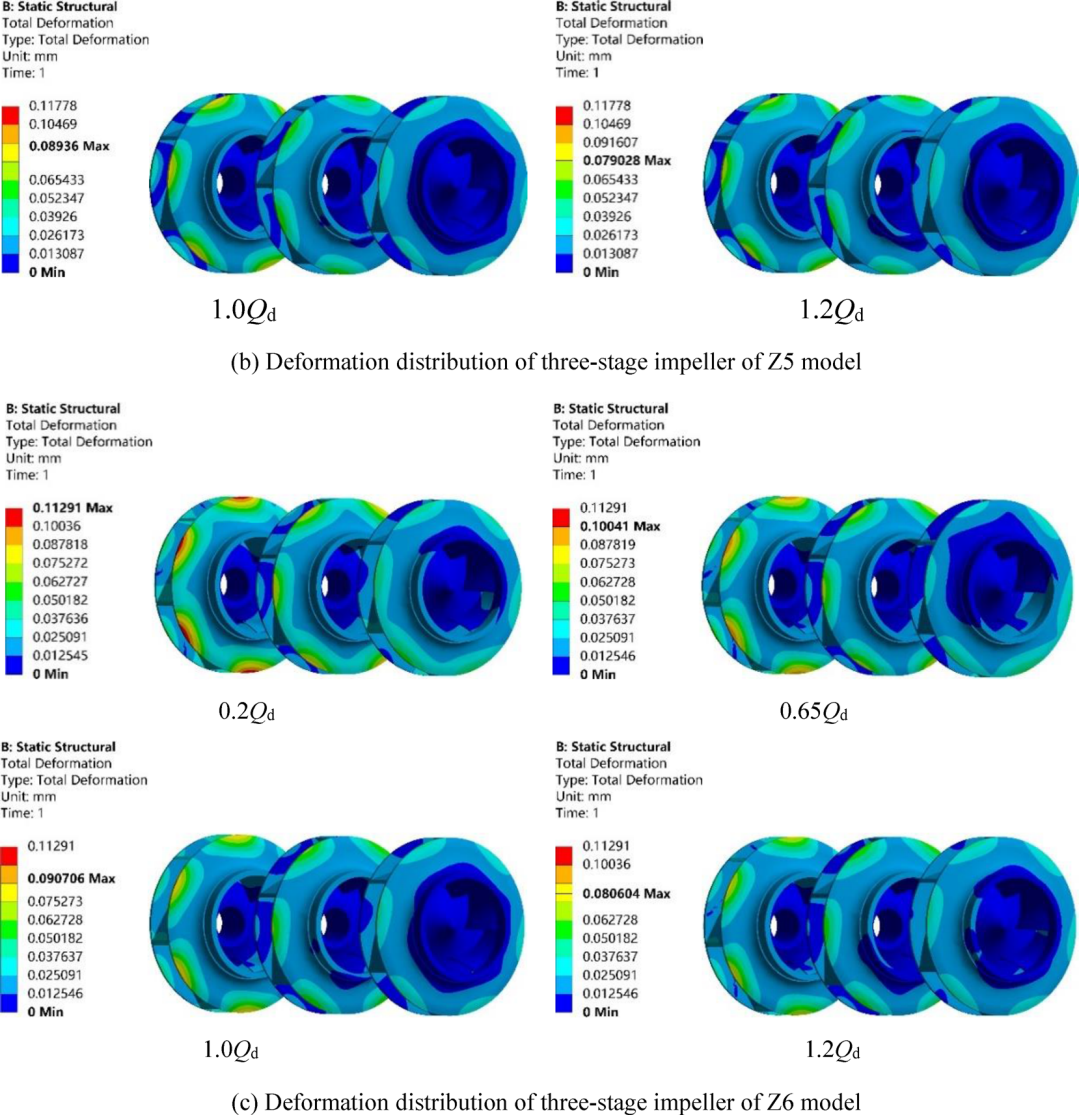
Deformation distribution of the three models under four working conditions.

Figure 11a–c show the deformation distributions of Z4, Z5 and Z6 models under the four working conditions of 0.2*Q*_d_, 0.65*Q*_d_, 1.0*Q*_d_ and 1.2*Q*_d_. The three impellers in the figures are called first-, second- and third-stage impeller from right to left, respectively (same below). According to the fluid–structure interaction theory, due to the action of fluid load on the impeller, the deformation of the rotor mainly occurs on the impeller. As shown in the figure, the deformation of the impeller increases as the number of impeller stage increases, and the maximum deformation occurs at the upper crown edge of the third-stage impeller. The reason is that the fluid gained energy due to the rotation of the impeller, and hence the more stages the fluid passes through the impeller, the more energy it obtains. Therefore, the fluid pressure on the first-stage impeller is the smallest, and as the number of the impeller stages increases, the fluid pressure on the impeller gradually increases. In addition, as the flow rate increases, the overall pressure distribution of the impellers becomes more uniform, because the outlet pressure of the impeller under high flow conditions is lower than that under low flow conditions. With the increase of the flow rate, the deformation of the impeller was continuously reduced and becomes relatively more uniform. Such trend is basically consistent with that of the fluid pressure inside the impeller.

### Rotor equivalent stress

**Figure 12:**
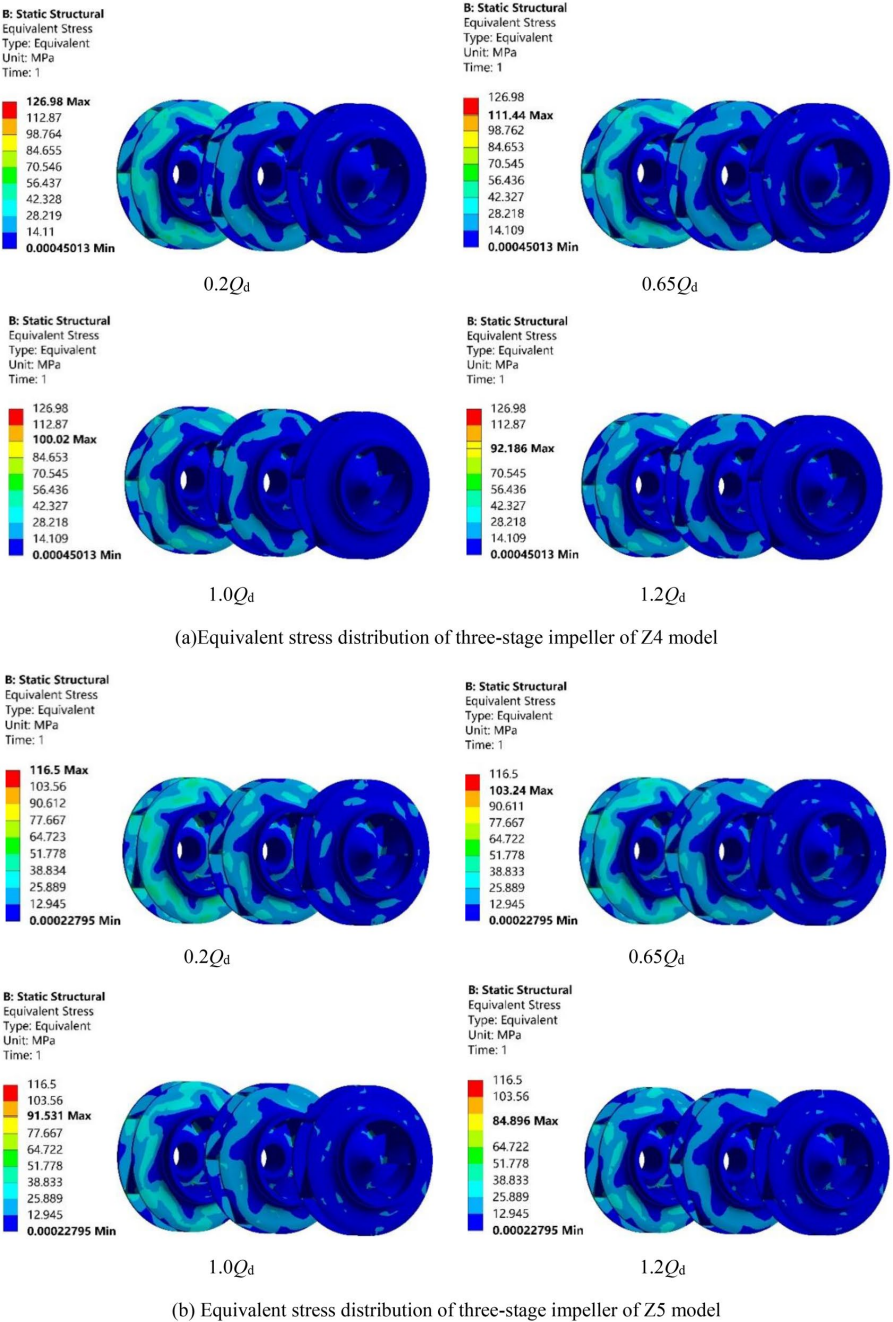

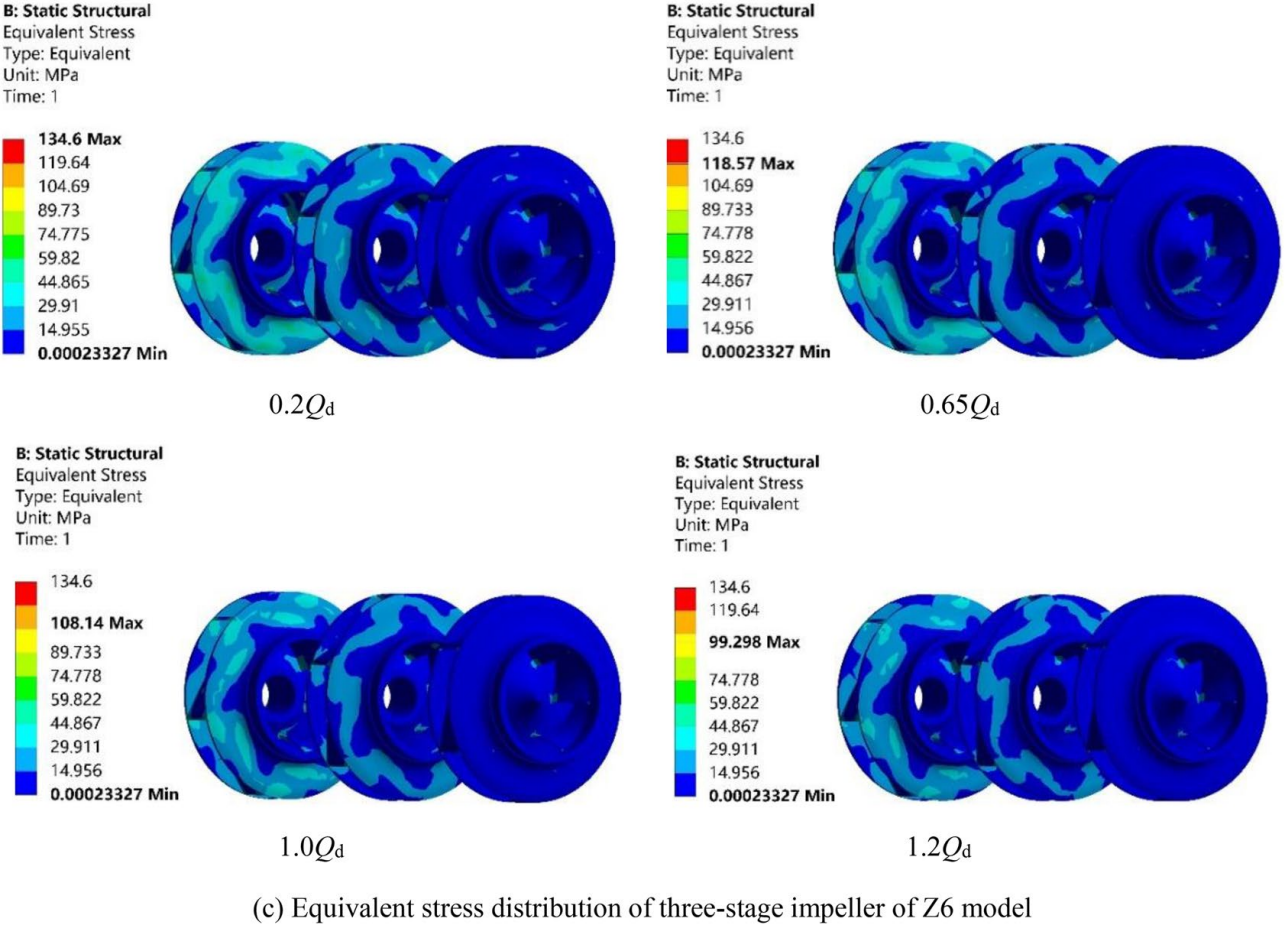
Equivalent stress distribution of the three models under four working conditions.

Figure 12a–c show the equivalent stress distribution of Z4, Z5 and Z6 models under the four working conditions of 0.2*Q*_d_, 0.65*Q*_d_, 1.0*Q*_d_ and 1.2*Q*_d_. Because the impeller is subjected to fluid–structure interaction, the equivalent stress of the rotor is mainly reflected in the impeller, the variation trend of which is the same as that of deformation. It can be seen from Fig. 12a that the maximum equivalent stress of the impellers of Z4 model under the four working conditions of 0.2*Q*_d_, 0.65*Q*_d_, 1.0*Q*_d_ and 1.2*Q*_d_ is 126.98 MPa, 111.44 MPa, 100.02 MPa and 92.186 MPa, respectively. The maximum value 126.98 MPa is found for the smallest flow condition 0.2*Q*_d_, which is still less than its allowable stress (which is 250 MPa as shown in “Model introduction” section), implying that the rotor material meets the strength requirements. Therefore, the structural strength of Z4 model meets the design requirements. Similarly, as shown in Fig. 12b,c, the maximum equivalent stress for Z5 model and Z6 model is 116.5 MPa and 134.6 MPa, respectively, both under 0.2*Q*_d_ condition, which is less than the allowable stress.

### Comparison of static characteristics of the three models

#### Comparison of change trends of total deformation

**Figure 13:**
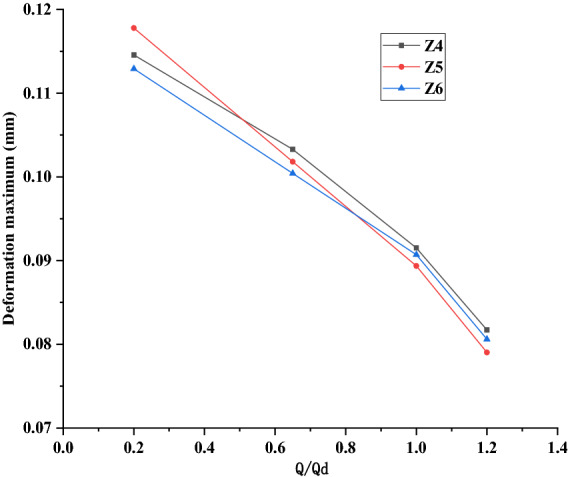
Variation of the maximum deformation of three models under four working conditions.

In order to intuitively reflect the change trends of the static parameters of three models, Fig. 13 shows the deformation maximum of the rotor under the four working conditions of 0.2*Q*_d_, 0.65*Q*_d_, 1.0*Q*_d_ and 1.2*Q*_d_ for the three models of Z4, Z5 and Z6. It can be seen from the figure that the change trends of the three models are basically the same, and the deformation is the largest under the small flow condition of 0.2*Q*_d_, which is because that the internal flow of the impeller is not uniform, and then some areas of the impeller would be subjected to larger force under the small flow condition. In addition, compared with Z4 and Z6 models, the deformation value of Z5 model (original model) represented by the red line has a greater downward trend with the increase of flow rate. Under rated conditions and beyond, the maximum deformation of Z5 model is always smaller than Z4 or Z6 model, which indicates that the structural design of Z5 model is more reasonable. However, the maximum deformation of Z4 and Z6 models is within a reasonable range (the rotor deformation grade is generally:10^–2^–10^−1^ mm), which also meets the requirements of the rotor structure design.

#### Comparison of change trends of equivalent stress

**Figure 14:**
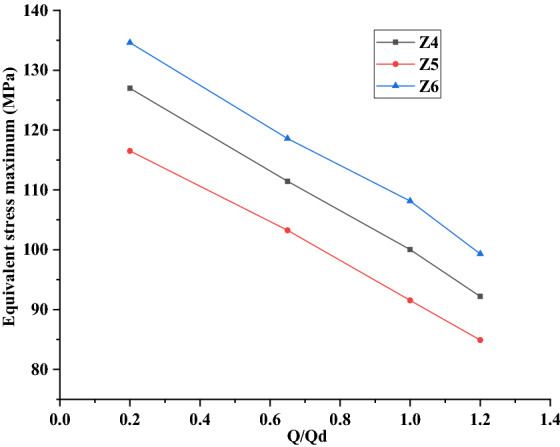
Equivalent stress maximum distribution of three models under four working conditions.

Figure 14 shows the maximum equivalent stress of Z4, Z5 and Z6 under the four working conditions of 0.2*Q*_d_, 0.65*Q*_d_, 1.0*Q*_d_ and 1.2*Q*_d_. It can be seen from the figure that the variation trends of the three models are basically the same, with the maximum value always occurs under the small flow condition of 0.2*Q*_d_. The reason of this phenomenon is also that the internal flow of the impeller is not uniform, and then some areas of the impeller would be subjected to larger force under the small flow condition. In addition, compared with Z4 and Z6 models, the equivalent stress value of Z5 model (original model) is the smallest, which indicates that the structural design of Z5 model is more reasonable. Though the maximum equivalent stress of Z4 and Z6 models is larger than Z5 model, it is still less than the allowable stress of the rotor material, which is also in line with the structural design safety requirements.

## Critical speed analysis of three models under different shaft lengths

### Rotor natural frequency analysis

**Figure 15:**
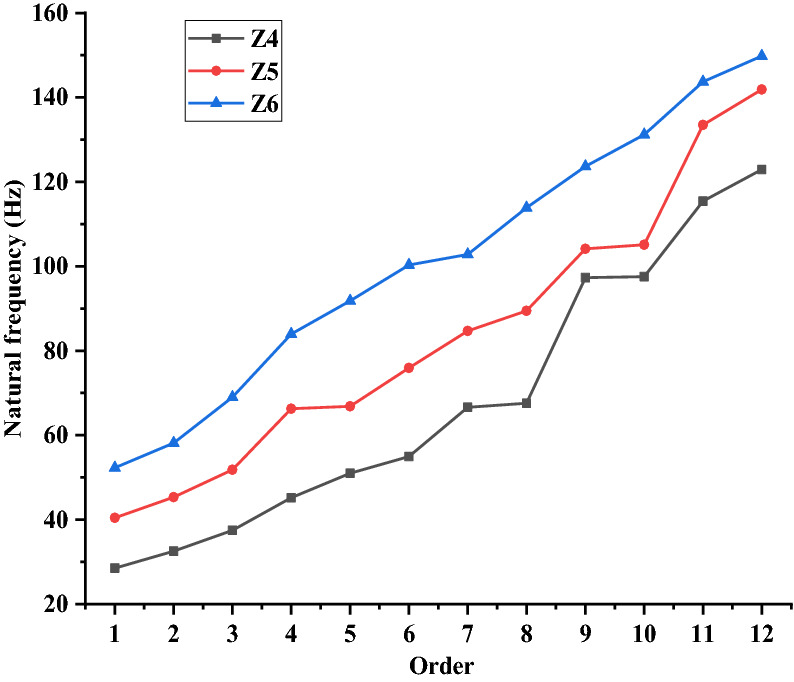
Natural frequency graphs of three models in the dry mode.

Figure 15 shows the first 12 natural frequencies of the three models of Z4, Z5 and Z6 in dry mode. Dry mode refers to the inherent mode of the structure in air, regardless of the influence of the surrounding fluid on the structure mode. Since the water mass of the impeller could be neglected, compared with the mass of the whole shafting rotor, and the pre-stress has little influence on the mode of the rotor, the paper chooses to analyze the inherent characteristics of the shafting rotor in the dry mode. As shown in Fig. 15, with the increase of the order, the natural frequency of the rotor generally presents an increasing trend. In addition, the natural frequency of Z6 model is the largest, and the natural frequency of Z4 model is the smallest under the same order. The reason of this phenomenon is that the longer the length of the single-segment intermediate shaft, the fewer the number of intermediate shafts, and the fewer couplings and bearing supports are needed. Thereby, the coupling and supporting rigidity of the entire shafting rotor are reduced, which directly reduces the natural frequency of the entire shafting rotor.

### Rotor vibration analysis

**Figure 16:**
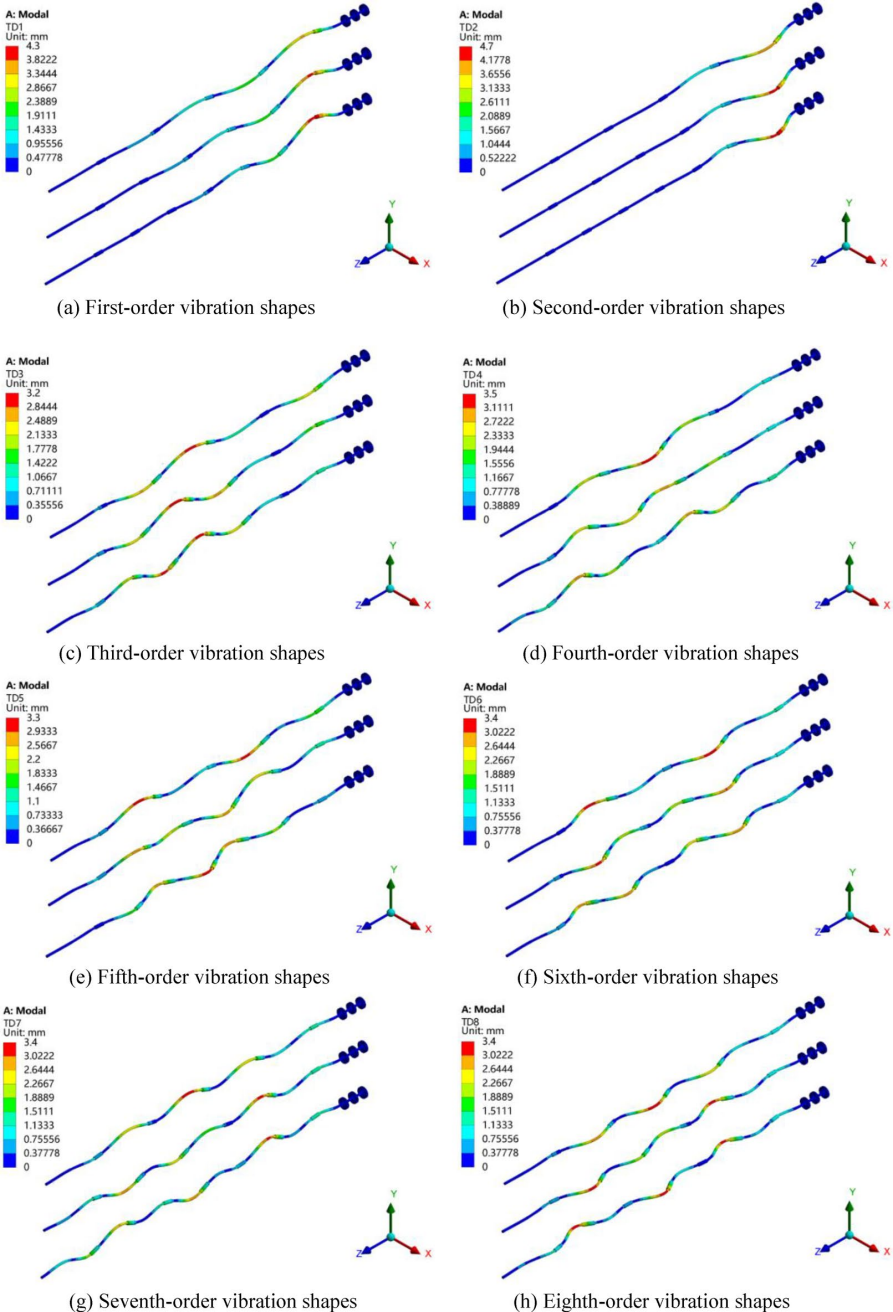

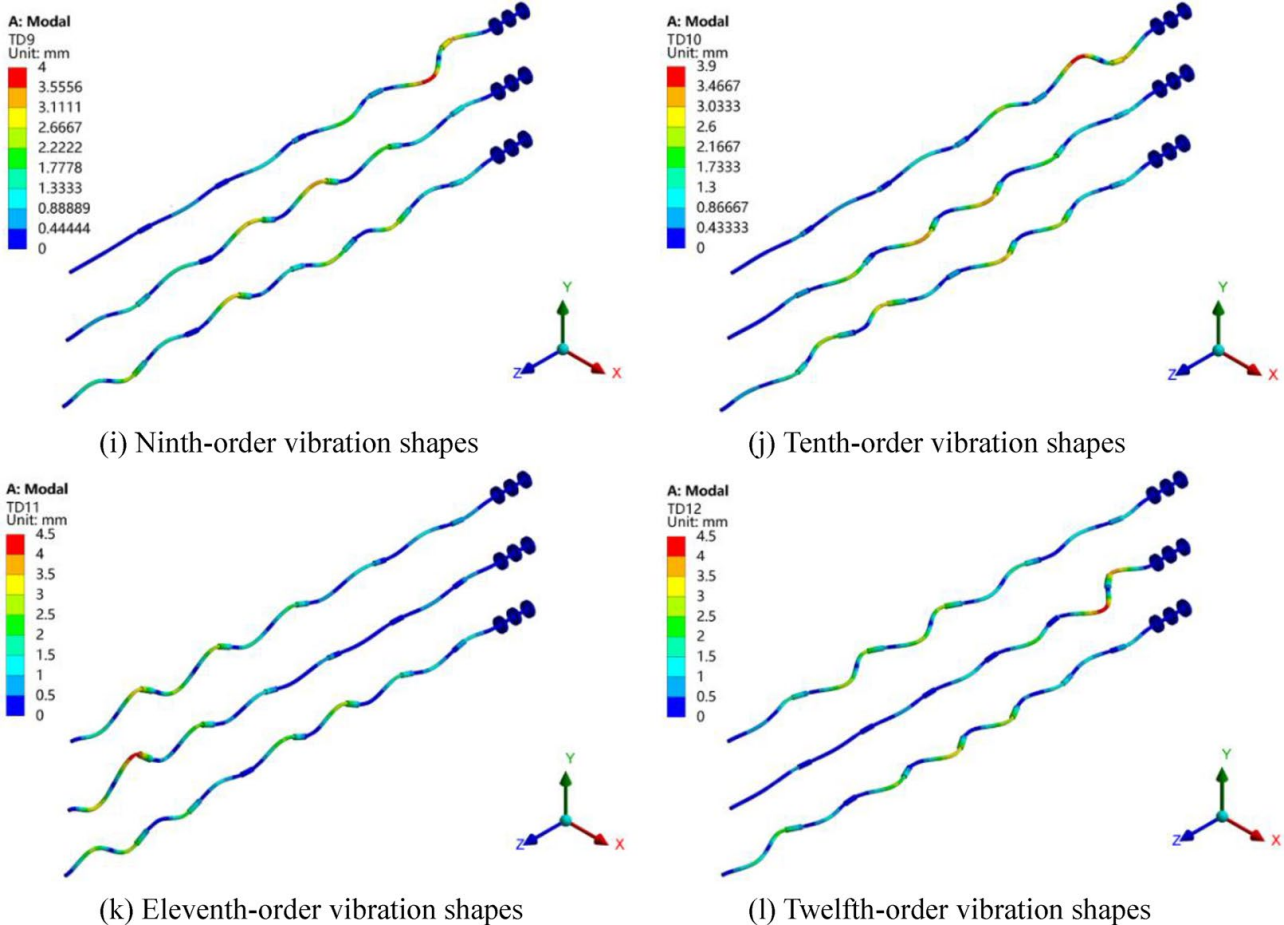
Vibration shapes of the three models.

The vibration shapes of the rotor can reflect the vibration and torsion amplitudes of each part of it, which is beneficial to find weaker part of the structure design. The first 12-order vibration shapes of three models of Z4, Z5 and Z6 are shown in Fig. 16. Here, the same order of vibration shapes of the three models is put together for analysis and the three models from top to bottom are Z4, Z5 and Z6 respectively. The coordinate system in the lower right corner reflects the actual orientation of the rotor, and the displacement magnification is 100. It can be seen in the figure that for three models, the first-order and second-order vibration shapes are the bending deformation and the largest deformation appears the middle of the first bearing and the first intermediate shaft. However, the higher the order, the more complex the vibration shape. For third-order vibration shapes and above, deformations of the rotor consist of transverse and torsional vibrations, and the blending positions basically occur on the intermediate shafts, which is due to the increase of the natural frequency and the critical speed of the whole shafting rotor system.

### Rotor critical speed analysis

Table 4 shows the critical speeds of the three models. In the table, “*WD*” represents the Whirl Direction, “*CS*” represents the Critical Speed, “BW” represents the Backward Whirl, and “FW” represents the Forward Whirl. According to the theoretical basis of rotor dynamics, the critical speeds corresponding to the “FW” are the actual critical speeds of the rotor. It can be seen from the table that the first six critical speeds of Z4 model are 1952.4 r/min, 2711.5 r/min, 3296.4 r/min, 4054.9 r/min, 5858.0 r/min and 7375.9 r/min, respectively. When the working speed of the rotor is lower than 0.8 times the first-order critical speed of it, the rotor can avoid the resonance^24^. Since the working speed of the VSLFP is 1485 r/min and 1485 is less than 0.8 $\times$ 1952.4, Z4 model structure meets the dynamic design requirement. Similarly, the first five critical speeds of Z5 model are 2719.6 r/min, 4555.7 r/min, 5368.5 r/min, 6311.4 r/min and 8513.8 r/min, respectively. Note that 1485 is less than 0.8 $\times$ 2719.6, indicating the structure of Z5 model meets the requirement of dynamic design. Similarly, for Z6 model, the first-order critical speed, 3488.9 r/min, is even greater, which indicates the structure of Z6 model meets the dynamic design requirement.

**Table 4:** Critical speeds of 3 models (r/min).

Order	*WD*_4_	*CS*_4_	*WD*_5_	*CS*_5_	*WD*_6_	*CS*_6_
1	BW	1709.5	BW	2425.5	BW	3137.5
2	FW	1952.4	FW	2719.6	FW	3488.9
3	BW	2247.4	BW	3108.0	BW	4139.9
4	FW	2711.5	BW	3976.6	BW	5037.4
5	BW	3058.4	BW	4009.2	FW	5509.4
6	FW	3296.4	FW	4555.7	FW	6018.7
7	BW	3995.3	BW	5081.6	BW	6167.5
8	FW	4054.9	FW	5368.5	FW	6831.8
9	BW	5832.3	BW	6244.0	BW	7418.1
10	FW	5858.0	FW	6311.4	FW	7872.1
11	BW	6924.1	BW	8008.3	BW	8619.9
12	FW	7375.9	FW	8513.8	FW	8991.5

**Figure 17:**
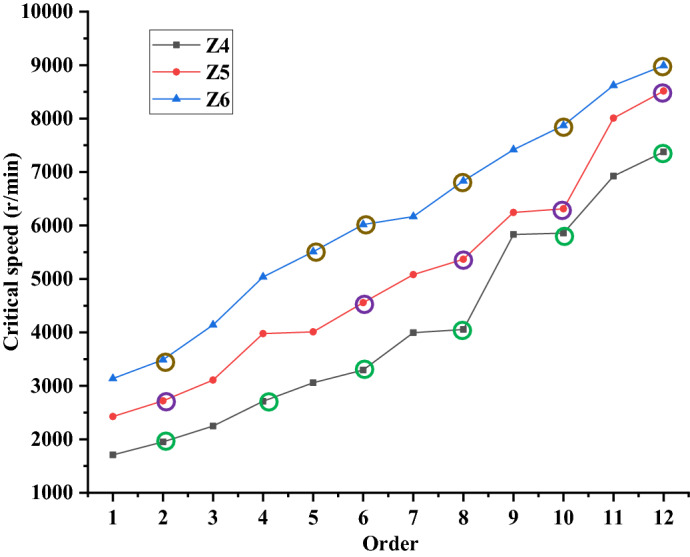
Comparison of the critical speeds on three models.

Figure 17 shows the critical speeds of Z4, Z5, and Z6 models in the first 12 modes. In the figure, the 6 green circles on the black curve represent the first 6-order critical speeds of Z4 model, the 5 purple circles on the red curve represent the first 5-order critical speeds of Z5 model, and the 6 brown circles on the blue curve represent the first 6-order critical speeds of Z6 model. In addition, the first-order critical speeds of three models all appear in the second-order mode. However, the second-order critical speed of Z4 model appears in the fourth-order mode, whereas that of Z6 model appears in the fifth-order mode, and that of Z5 model appears in the sixth-order mode. This indicates that the second-order resonance of Z5 model (original model) is delayed, compared with Z4 and Z6 models. Furthermore, it can be seen from the figure that the critical speed of Z6 model is the largest, and the critical speed of Z4 model is the smallest. The reason for this phenomenon is the same as in “Rotor natural frequency analysis” section.

### Optimal model selection

According to the stress check of the impeller, the critical speed check of the shafting rotor and so on, all three models (Z4 model, Z5 model and Z6 model) meet the requirements of the rotor structure design and the dynamic design. However, in actual engineering project, the VLSFP, which is connected with more shaft segments, needs to take more time and the labor cost when it is installed and disassembled many times before and after the test or operation. To sum up, the paper selects the Z4 model to be the optimal model and provides a theoretical support for the subsequent design optimization of the vertical long shaft fire pump.

## Conclusions

The paper selected XBC18-178-240LC3 VLSFP as the research object, using modeling software to design three models (Z4 model, Z5 model and Z6 model) connected by different length and number of the shaft section under the same total length of the intermediate shafts, and then the rotor’s strength and critical speed of three models were analyzed and checked via CFD simulation and Workbench software. The main conclusions were as follows:

1. With the increase of the flow rate, the deformation and equivalent stress of the impeller was continuously reduced and became more uniform.
2. Through the strength check of the impeller, maximum equivalent stress of the three models was less than the allowable stress of the rotor material, which indicated the structural design of them met the safety requirement.
3. Through the critical speed check of the shafting rotor, the working speed of the VLSFP was lower than 0.8 times the first-order critical speed of the three models, which indicated the rotor can avoid the resonance and the structure of the three models met the dynamic design requirement.
4. According to the stress check of the impeller and the critical speed check of the shafting rotor, combining the time and labor cost when the VLSFP was installed and disassembled many times before and after the test or operation, the paper selected the Z4 model to be the optimal model, which can provide a theoretical support for the subsequent design optimization of the vertical long shaft fire pump.
5. The paper lacked the experimental verification of the dynamics. Due to the complex structure and large volume of the VLSFP, there was no corresponding test site at present. Therefore, in the subsequent research, scaling down the VLSFP model would be considered, and then the rotor dynamics test would be carried out.

## Data Availability

The datasets generated or analyzed during the current study are available from the corresponding author on reasonable request.
